# Supplementary material for: No Evidence of Benefits of Host Nano-Carbon Materials for Practical Lithium Anode-Free Cells
Source: Nanomaterials (Basel). 2022 Apr 20;12(9):1413. doi: 10.3390/nano12091413 (PMC9101068; doi:10.3390/nano12091413)
Supplement: Supplementary file 1 [file nanomaterials-12-01413-s001.zip › 220414-Supplementary Materials-01.pdf]

Supplementary Materials

No Evidence of Benefits of Host Nano-Carbon Materials for Practical Lithium Anode-Free Cells

Bingxin Zhou, Baizeng Fang, Ivan Stoševski, Arman Bonakdarpour and David P. Wilkinson *

Department of Chemical & Biological Engineering, the Clean Energy Research Center, University of British Columbia, 2360 East Mall, Vancouver, BC V6T 1Z3, Canada; bxzhou@mail.ubc.ca (B.Z.); bfang@chbe.ubc.ca (B.F.); ivan.stosevski@ubc.ca (I.S.); arman@chbe.ubc.ca (A.B.)

* Correspondence: dwilkinson@chbe.ubc.ca (D.P.W.)

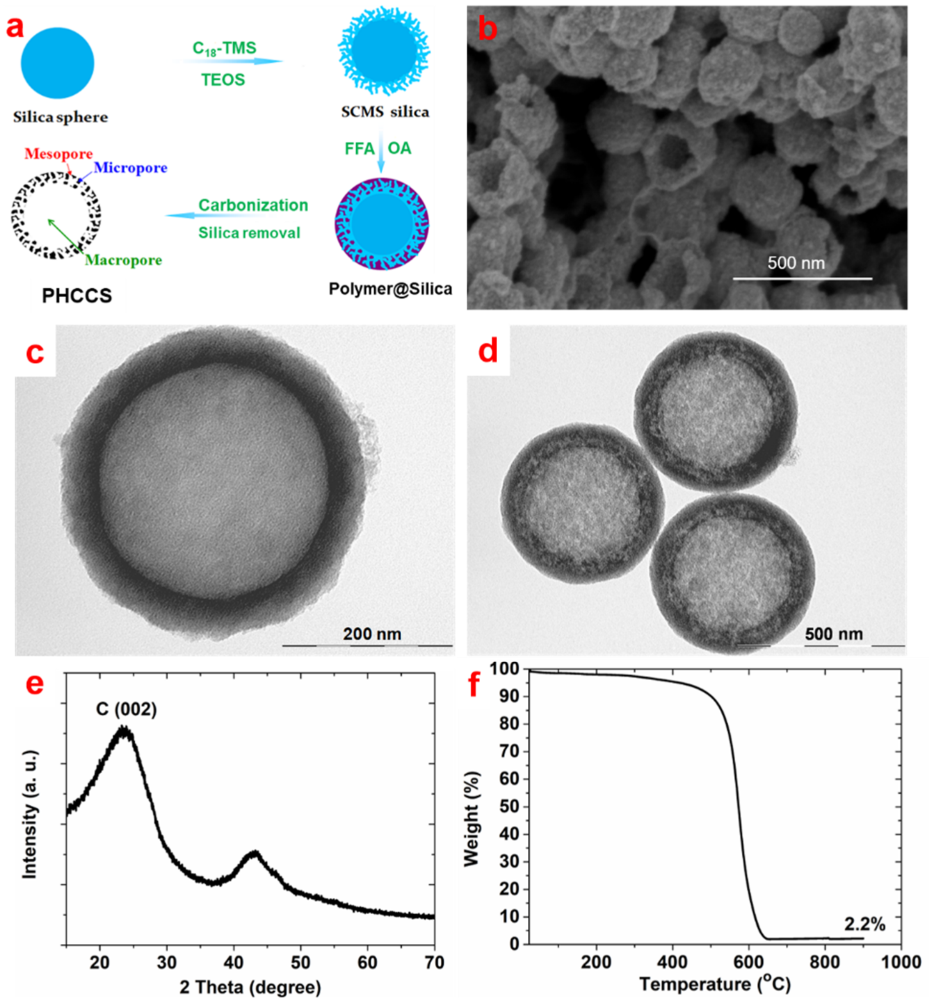

Figure S1. (**a**) Schematic illustration for the synthesis of PHCCSs; (**b**) SEM image of the PHCCSs with a core diameter of 110 nm and a shell thickness of 60 nm; TEM images for (**c**) PHCCSs (270 nm : 55 nm) and (**d**) PHCCSs (360 nm : 55 nm); (**e**) XRD pattern and (**f**) TGA plot for the PHCCSs (110 nm : 60 nm).

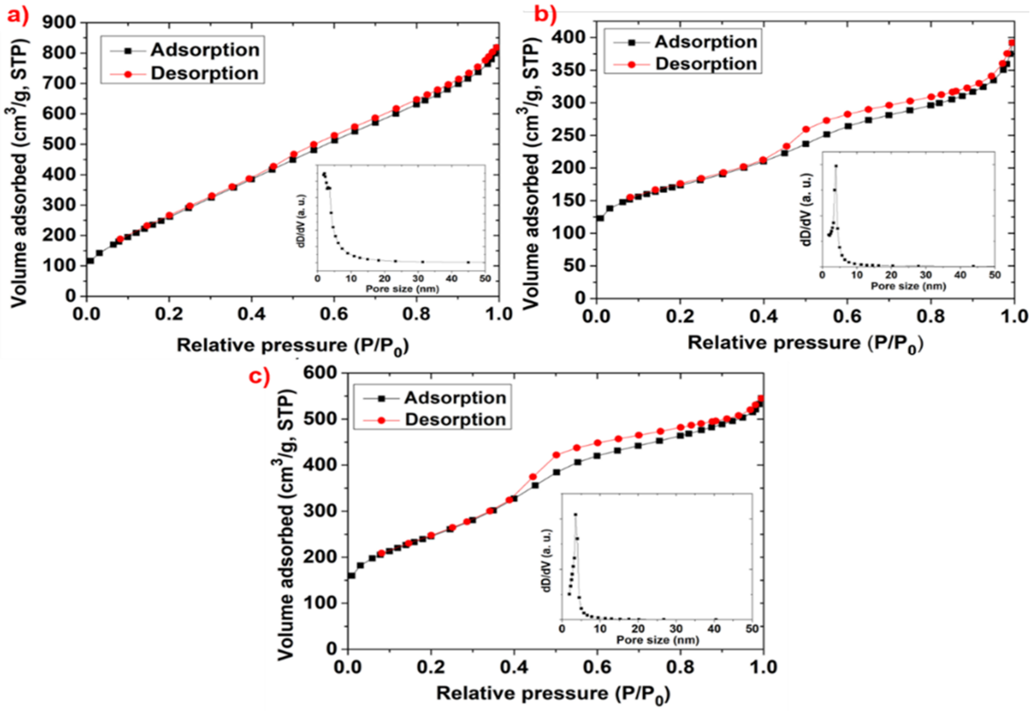

Figure S2. N_2 adsorption-desorption isotherms for the PHCCSs with various core diameters: (**a**) 110 nm; (**b**) 270 nm; and (**c**) 360 nm.

Table S1. Parameters derived from the N_2 adsorption-desorption isotherms for various PHCCSs

**Sample**	**Area (m^2/g)**	**Pore volume (m^3/g)**	**Micropore volume (m^3/g)**	**Pore diameter (nm)**
110 nm : 60 nm	1072	1.269	0.021	4.1
270 nm : 55 nm	581	0.608	0.098	4.6
360 nm : 55 nm	865	0.845	0.060	3.8

Table S2. Capacity calculation parameters of PHCCSs (110 nm : 60 nm)

**Parameter (Unit)**	**Quantity**
Diameter of hollow core, D_h (nm)	110
Shell thickness, T_S (nm)	60
Thickness of the carbon layer T (μm)	15
Surface area of the electrode, $A_{electrode}$ (cm^2)	1.767
Capacity of cathode (mAh)	3.133
Active material percentage, P_a	0.84
The six most densely packed/space utilization	0.74
Hollow core volume, V_H(cm^3)	0.00018
Volume of carbon coating, V_c(cm^3)	0.0026
Capacity of hollow core, $Capacity_{core}$ (mAh)	0.372
Volumetric Capacity based on carbon coating volume, V_{cv} (mAh/cm^3)	140.16

Equations used to calculate the volumetric capacity of hollow core:

$$V_H(cm^3) = 0.74 \times P_a \times \frac{(0.5D_h)^3}{(0.5D_h+T_S)^3} \times T \times A_{electrode} \div 10000 \quad (1)$$

$$Capacity_{core}(mAh) = V_H \times 3860\,\frac{mAh}{g} \times 0.534\,\frac{g}{cm^3} \quad (2)$$

$$V_{CV}\,(\frac{mAh}{cm^3}) = \frac{Capacity_{core}}{A_{electrode}T} \times 10000 \quad (3)$$
